# Leveraging Prior Knowledge in a Hybrid Network for Multimodal Brain Tumor Segmentation

**DOI:** 10.3390/s25154740

**Published:** 2025-08-01

**Authors:** Gangyi Zhou, Xiaowei Li, Hongran Zeng, Chongyang Zhang, Guohang Wu, Wuxiang Zhao

**Affiliations:** College of Electronics and Information Engineering, Sichuan University, Chengdu 610017, China; zhougangyi@stu.scu.edu.cn (G.Z.);

**Keywords:** medical segmentation, multimodal, brain tumor image, attention mechanism, prior knowledge

## Abstract

Recent advancements in deep learning have significantly enhanced brain tumor segmentation from MRI data, providing valuable support for clinical diagnosis and treatment planning. However, challenges persist in effectively integrating prior medical knowledge, capturing global multimodal features, and accurately delineating tumor boundaries. To address these challenges, the Hybrid Network for Multimodal Brain Tumor Segmentation (HN-MBTS) is proposed, which incorporates prior medical knowledge to refine feature extraction and boundary precision. Key innovations include the Two-Branch, Two-Model Attention (TB-TMA) module for efficient multimodal feature fusion, the Linear Attention Mamba (LAM) module for robust multi-scale feature modeling, and the Residual Attention (RA) module for enhanced boundary refinement. Experimental results demonstrate that this method significantly outperforms existing approaches. On the BraT2020 and BraT2023 datasets, the method achieved average Dice scores of 87.66% and 88.07%, respectively. These results confirm the superior segmentation accuracy and efficiency of the approach, highlighting its potential to provide valuable assistance in clinical settings.

## 1. Introduction

Brain tumors, abnormal tissue growths within the cranial cavity, can be either benign or malignant. They may originate from brain cells or metastasize from cancer cells in other parts of the body [1]. The pathogenesis of brain tumors is complex, and their growth can increase intracranial pressure, causing symptoms such as headaches, nausea, and vomiting. Additionally, they can affect brain regions responsible for cognitive, sensory, and motor functions, leading to cognitive impairment, sensory loss, and motor dysfunction. These characteristics make brain tumors a significant health concern. Although brain tumors are not the most common type of cancer globally, their unique anatomical location and complex biological characteristics often result in a marked reduction in patient survival rates, increased treatment difficulty, and substantial impacts on quality of life [2].

**Figure 1:**
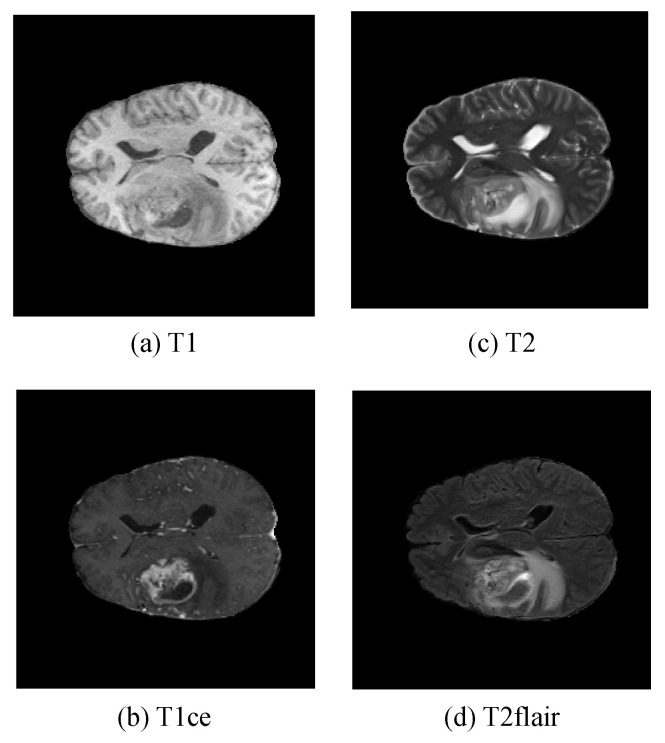
Multimodal morphology of medical images of brain tumors.

Magnetic Resonance Imaging (MRI) [3] is a cutting-edge medical imaging technology widely used in brain tumor diagnosis due to its exceptional soft tissue contrast resolution. MRI provides detailed visualizations of tumor size and shape, as well as relationships between the tumor and surrounding brain tissues, aiding physicians in assessing tumor nature and location and offering precise navigational information for preoperative planning. MRI typically includes four modalities [4]: T1-weighted, contrast-enhanced T1-weighted, T2-weighted, and T2-weighted Fluid-Attenuated Inversion Recovery (FLAIR) images, as shown in Figure 1. However, the tumor regions in these multimodal images often exhibit complex morphological and structural features, particularly in cases with small lesions and indistinct boundaries, posing challenges for segmentation accuracy.

In recent years, with the advancement of artificial intelligence, particularly deep learning, significant progress has been made in brain tumor segmentation. Deep learning models such as U-Net [5] have demonstrated remarkable capability in medical image analysis, accurately segmenting brain tumors’ location and shape from complex MRI data. This technology plays a crucial role in assisting clinicians with diagnosis and treatment planning. Brain tumor segmentation not only enhances diagnostic precision but also significantly improves workflow efficiency. Automating image processing alleviates the workload on physicians, allowing them to dedicate more time to patient care and decision making.

Despite these advancements, existing MRI-based brain tumor segmentation methods face numerous challenges. Firstly, many current approaches fail to fully leverage prior medical knowledge, limiting segmentation accuracy. Incorporating and utilizing such prior knowledge can effectively enhance segmentation precision. Secondly, in multimodal medical segmentation tasks, existing methods often struggle to capture global information from multimodal features, making it difficult to fully integrate the rich information across different modalities. Additionally, many algorithms lack the ability to accurately capture fine details of tumor boundaries, especially when dealing with blurred edges or small lesions, resulting in less refined segmentation outcomes.

To tackle the challenges of multimodal brain tumor segmentation, this paper proposes a novel approach named Hybrid Network for Multimodal Brain Tumor Segmentation (HN-MBTS). This method leverages prior medical knowledge to enhance global feature extraction from multimodal data and improve the precision of boundary detail segmentation. The main contributions of this work are outlined as follows:

- A Two-Branch, Two-Model Attention (TB-TMA) module is introduced, utilizing prior medical knowledge to reorganize multimodal inputs based on clinical relevance. Single-modality features and cross-modality information are effectively modeled and fused through independent branches and cross-attention mechanisms, significantly enhancing cross-modal feature representation.
- A Linear Attention Mamba (LAM) module is proposed, incorporating a linear attention mechanism to improve the computational efficiency of multi-scale feature modeling. This module is designed to enhance the network’s adaptability to large-scale multimodal data, ensuring effective learning of complex features.
- A Residual Attention (RA) module is developed to capture fine details in segmentation boundary regions. Multiple attention units are stacked, and a residual structure is utilized to further improve segmentation accuracy, providing precise feature extraction for boundary refinement.

## 2. Related Work

### 2.1. CNN-Based Medical Brain Tumor Segmentation

CNN-based models have demonstrated impressive performance in brain tumor segmentation [6]. Due to computational and memory constraints, early CNN-based methods [7,8] focused on segmenting 2D MRI slices. Subsequently, a growing number of three-dimensional brain tumor segmentation (BTS) models have been proposed. 3D U-Net [9], a fully automated organ segmentation model based on 3D convolutional neural networks, employs 3D convolutions to process 3D images, preserving spatial information and enhancing segmentation accuracy and robustness. The model integrates features from both encoder and decoder structures, following the U-Net architecture. V-Net [10] extends the 3D convolution framework by incorporating the U-Net structure, offering a network architecture for 3D image segmentation. It also introduces the Dice coefficient loss function to address the class imbalance problem in segmentation samples. Ramin et al. [11] proposed an automatic and robust brain tumor segmentation framework using an optimized convolutional neural network (CNN). The weights and biases of the network are fine-tuned through the Improved Chimp Optimization Algorithm (IChOA) to handle multimodal data. AD-Net [12] introduces an automatic weighted dilated convolutional network that learns multimodal brain tumor features through channel-wise feature separation. A novel method proposed in [13] utilizes two sub-networks within the projected cascaded convolutional neural network framework: the Tumor Localization Network (TLN) and the LSIS-based Intra-tumor Segmentation Network (LITSN), effectively segmenting tumors with high-grade gliomas from 3D MRI data.

CNN-based brain tumor segmentation models have made significant strides in the field of medical image segmentation, evolving from early 2D segmentation models to more sophisticated 3D convolutional neural networks. These models have progressively improved segmentation accuracy and robustness by integrating various network architectures and optimization techniques. However, despite their effectiveness in handling single-modality data, fully leveraging multimodal information remains an open challenge.

### 2.2. Transformer-Based Medical Brain Tumor Segmentation

An increasing number of medical image segmentation studies have explored network architectures based on Vision Transformers (ViTs). UNETR [14] employs a Transformer in the encoder to extract features from 3D brain tumor images, connecting features at different resolutions to the decoder via skip connections, thereby capturing global contextual information across multiple scales. Similarly, Swin UNETR [15] replaces standard convolutions in the encoder with the Swin Transformer [16], leveraging its hierarchical structure. Li et al. [17] introduced a hybrid approach by integrating a Transformer at the bottleneck of an encoder–decoder architecture to combine the advantages of both 3D convolutions and Transformers. Peiris et al. [18] proposed a volumetric Transformer for 3D tumor segmentation, utilizing the self-attention mechanism of Transformers in the encoder to capture both local and global features. The decoder incorporates self-attention and cross-attention mechanisms to capture fine-grained features.

The Transformer architecture is becoming a research focal point in the field of medical image segmentation, demonstrating significant potential in 3D brain tumor segmentation due to its powerful global feature extraction capabilities. However, existing Transformer-based methods still face challenges in capturing fine details and effectively integrating multimodal data.

### 2.3. Multimodal Medical Segmentation Methods

Zhang [19] proposed the Multimodal Contrastive Domain Sharing (Multi-ConDoS) GAN network to achieve effective multimodal, contrastive, self-supervised medical image segmentation. This approach leverages multimodal medical images to learn more comprehensive object features through multimodal contrastive learning. To address the fusion of Positron Emission Tomography (PET) and Computed Tomography (CT) data, Marinov [20] introduced Mirror U-Net, which decomposes multimodal representations into modality-specific decoder branches and an auxiliary multimodal decoder, replacing traditional fusion methods with multimodal fission. Pandey [21] presented a multimodal medical segmentation method combining YOLOv8 and SAM, enabling the segmentation of regions of interest (ROIs) across various medical imaging datasets, including ultrasound readings, CT scans, and X-ray images. Andrade [22] described a unified framework for various Transformer-based architectures, exploring the performance differences between single-path and multi-path encoders and examining the impact of multi-stream interaction on multimodal Transformers.

Multimodal medical segmentation methods aim to enhance segmentation performance by integrating information from different modalities. However, current approaches still face limitations in capturing both global information and fine details of multimodal features. The hybrid network proposed in this paper leverages prior knowledge to organically combine information from different modalities, guiding the model to achieve more precise and detailed brain tumor segmentation.

## 3. Method

**Figure 2:**
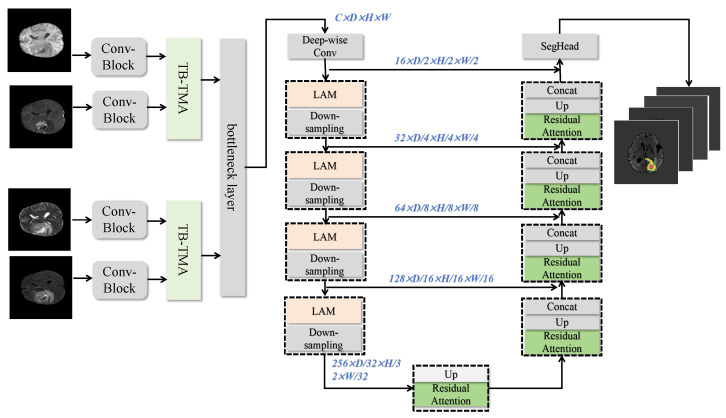
Structural diagram of the Hybrid Network for Multimodal Brain Tumor Segmentation algorithm.

This paper introduces HN-MBTS (Hybrid Network for Multimodal Brain Tumor Segmentation), a hybrid network designed to efficient process multimodal brain tumor data to achieve precise segmentation. HN-MBTS is built on an encoder–decoder structure and incorporates three core innovative modules: two-branch, two-model attention (TB-TMA), linear attention Mamba (LAM), and residual attention (RA). The architecture is illustrated in Figure 2.

First, the TB-TMA module leverages prior medical knowledge to reorganize multimodal inputs into two clinically relevant groups. Each group is processed through an independent branch, where a self-attention module models long-range dependencies and extracts local features for single-mode data. A cross-attention module then integrates information across modalities, effectively capturing inter-modality correlations and significantly enhancing cross-modality feature representation. Next, features are passed to the LAM module, which introduces a linear attention mechanism to improve the computational efficiency of multi-scale feature modeling. This module efficiently models multimodal data by incorporating prior knowledge, enhancing the network’s adaptability to large-scale data while maintaining robust learning capabilities for complex multimodal features. Finally, the RA module combines prior medical knowledge with multiple stacked attention units to further enhance boundary detail capture. The RA module flexibly captures discriminative features across different scales and dimensions. Particularly in the boundary regions, the residual structure improves segmentation precision by effectively modeling detailed features.

In the decoding phase, HN-MBTS progressively restores spatial resolution and aggregates multi-scale features from the encoding phase through attention mechanisms. This leads to optimized feature fusion and ultimately generates high-precision brain tumor segmentation results. This network design, guided by prior medical knowledge, integrates efficient multimodal feature modeling with precise boundary capture, significantly improving performance in brain tumor segmentation tasks.

### 3.1. TB-TMA Module

In multimodal brain tumor segmentation tasks, different modalities often contain complementary information. However, directly fusing multimodal data can introduce redundancy and noise, adversely affecting segmentation accuracy. Thus, effectively extracting and integrating multimodal features becomes a critical challenge. Inspired by radiologists’ approach to evaluating brain tumor MRI images, this paper leverages prior knowledge to enable the model to learn spatial and structural associations between related sequences. The assessment of brain tumors typically relies on the combined interpretation of different modalities. For instance, T1-weighted (T1) and T1-weighted with contrast enhancement (T1Gd) imaging are used to assess blood–brain barrier disruption and define the tumor core, whereas T2-weighted (T2) and T2 FLAIR (fluid-attenuated inversion recovery) imaging are employed to detect free water and distinguish tumor necrosis from vasogenic edema.

Based on this clinical knowledge, the four image modalities—$X_{T1},X_{T1\mathrm{Gd}},X_{T2}$, and $X_{T2\mathrm{FLAIR}}$ are reorganized into two groups of related modalities: (1) $X_{T1}$ and $X_{T1\mathrm{Gd}}$ and (2) $X_{T2}$ and $X_{T2\mathrm{FLAIR}}$. Each group is processed independently within the model.

Unlike existing brain tumor segmentation models that concatenate all input modalities and feed them into the model simultaneously, this reorganization allows the model to better capture the correlations between modalities, leading to more accurate tumor region segmentation. The formulation is as expressed as follows:

(1) $$S=f_{\mathrm{ours}}\left(\theta ,\left(\left\{X_{T1},X_{T1\mathrm{Gd}}\right\},\left\{X_{T2},X_{T2\mathrm{FLAIR}}\right\}\right)\right)$$

**Figure 3:**
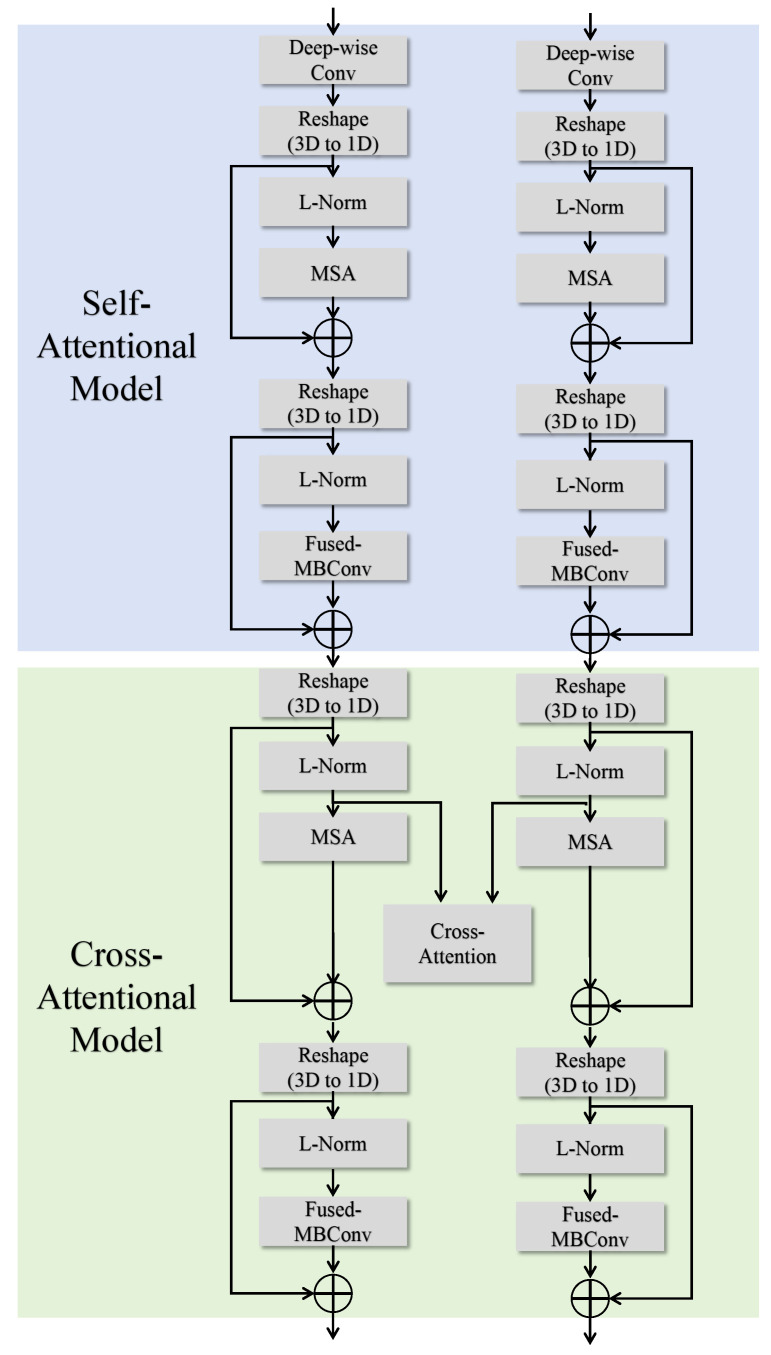
Structural diagram of the TB-TMA module.

**Figure 4:**
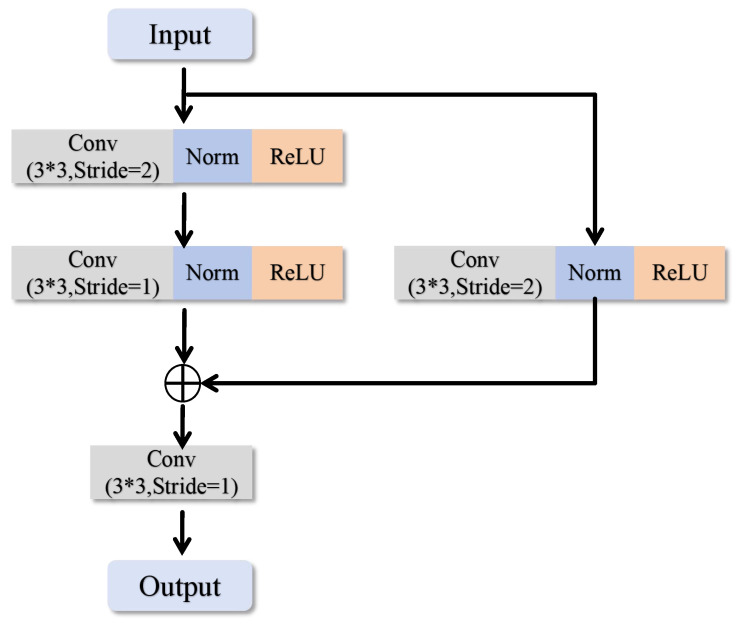
Structural diagram of the conv block.

To fully exploit the complementary information between different modalities in multimodal brain tumor segmentation tasks, this paper introduces the two-branch, two-model attention (TB-TMA) module, as illustrated in Figure 3. The design of the TB-TMA module is inspired by clinical knowledge, particularly the combined interpretation of different modalities in radiology. By dividing the input data into two independent branches and extracting features for each modality separately, TB-TMA effectively captures the relationships and complementarities between modalities. The multimodal brain tumor images are divided into two branches, which first pass through a conv block. The conv block is a convolutional module designed for the initial extraction of features from the input images, as shown in Figure 4. Its primary function is to transform the raw input data into feature representations more suitable for subsequent extraction and processing.

The TB-TMA module consists of two main components: the self-attentional model and the cross-attentional model. The self-attentional model focuses on modeling long-range dependencies and extracting local features within each modality independently. In contrast, the cross-attentional model leverages cross-modal information fusion to enhance the representation of features by capturing the correlations between different modalities. This dual attention mechanism ensures that the model can effectively utilize the complementary information provided by the multimodal data, leading to more accurate segmentation of brain tumor regions.

#### 3.1.1. Self-Attentional Model

The self-attentional model is designed to independently extract modality-specific features within each branch. It begins with deep convolution operations on the input images, transforming them into one-dimensional representations. Subsequently, a multi-head self-attention (MSA) mechanism [23] is employed to capture global features by computing self-attention across different positions. These features are then normalized using Layer Normalization (L-Norm). Finally, local features are further extracted using the Fused-MBConv module [24] to enhance the feature representation capabilities. The process is described by the following formulas:

(2) $$\left\{\begin{array}{c} F_{\mathrm{Tl}}^{l}=\mathrm{MSA}\left(\mathrm{LN}\left(F_{\mathrm{Tl}}^{l-1}\right)\right)+F_{\mathrm{Tl}}^{l-1}\\ F_{\mathrm{Tl}}^{l+1}=\mathrm{Fused}-\mathrm{MBConv}\left(\mathrm{LN}\left(F_{\mathrm{Tl}}^{l}\right)\right)+F_{\mathrm{Tl}}^{l} \end{array}\right.$$

(3) $$\left.\left\{\begin{array}{c} F_{\mathrm{TlGd}}^{l}=\mathrm{MSA}\left(\mathrm{LN}\left(F_{\mathrm{TlGd}}^{l-1}\right)\right)+F_{\mathrm{TlGd}}^{l-1}\\ F_{\mathrm{TlGd}}^{l+1}=\mathrm{Fused}-\mathrm{MBConv}\left(\mathrm{LN}\left(F_{\mathrm{TlGd}}^{l}\right)\right)+F_{\mathrm{TlGd}}^{l} \end{array}\right.\right.$$

Here, $\mathrm{MSA}\left(\cdot \right)$ denotes the multi-head self-attention module, which captures global feature dependencies by computing self-attention across different positions. Fused-$\mathrm{MBConv}\left(\cdot \right)$ is typically used for efficient feature extraction, leveraging depthwise separable convolutions to reduce computational complexity.

#### 3.1.2. Cross-Attentional Model

Building upon the features extracted by the self-attentional model, the cross-attentional model introduces a cross-attention mechanism to capture complementary information between different modalities. The features from both branches are normalized, then interact through a cross-attention layer to generate a fused feature representation. After applying layer normalization (LN) to the input feature maps, the standard multi-head self-attention module computes the relationships between features. The outputs of the cross-modality self-attention for Tl and TlGd are added to the self-attention outputs and the original input features, forming the fused features:

(4) $$M_{\mathrm{Tl}},M_{\mathrm{TlGd}}=\mathrm{CM}-\mathrm{MSA}\left(\mathrm{LN}\left(F_{\mathrm{Tl}}^{l+1}\right),\mathrm{LN}\left(F_{\mathrm{TlGd}}^{l+1}\right)\right)$$

(5) $$M_{\mathrm{Tl}}=\mathrm{SoftMax}\left(\frac{Q_{\mathrm{Tl}}K_{\mathrm{TlGd}}^{T}}{\sqrt{d}}+B\right)V_{\mathrm{TlGd}}$$

(6) $$M_{\mathrm{TlGd}}=\mathrm{SoftMax}\left(\frac{Q_{\mathrm{TlGd}}K_{\mathrm{Tl}}^{T}}{\sqrt{d}}+B\right)V_{\mathrm{Tl}}$$

The fused feature maps are normalized again and further refined using the Fused-MBConv module. Finally, the output features are connected with the input features through a residual connection, producing the final feature outputs $F_{\mathrm{Tl}}^{l+3}$ and $F_{\mathrm{TlGd}}^{l+3}$. After processing through the TB-TMA module, the task of the bottleneck layer is to concatenate the features from the four different modalities and further compress and fuse these concatenated features to effectively extract and express multimodal information:

(7) $$F_{\mathrm{concat}}=\mathrm{Concat}\left(F_{T1}^{l+3},F_{\mathrm{TlGd}}^{l+3},F_{T2}^{l+3},F_{T2\mathrm{FLAIR}}^{l+3}\right)$$

A 1×1 convolution is then applied to the concatenated features for channel compression:

(8) $$F_{\mathrm{bottleneck}}=\mathrm{Conv}1x1\left(F_{\mathrm{concat}}\right)$$

### 3.2. LAM Module

Mamba [25] is an innovative deep learning architecture designed to efficiently handle long sequence data. It achieves this through the Selective State Space Model (SSM), which captures temporal dependencies in sequential data and dynamically adjusts the parameters of the state space using a selective mechanism to manage long sequences efficiently. The model leverages a multi-head mechanism to process features from different modalities or scales in parallel and employs a gating mechanism for selective feature fusion, thereby enhancing performance in visual tasks. The core formula is expressed as follows:

(9) $$h_{t+1}=\sum_{head=1}^{H}\alpha^{\left(head\right)}\left(A_{d}^{\left(head\right)}h_{t}+B_{d}^{\left(head\right)}x_{t}\right)$$

Here, $h_{t+1}$ represents the updated feature state. $A_{d}^{\left(\mathrm{head}\right)}$ and $B_{d}^{\left(\mathrm{head}\right)}$ are the feature transition and input mapping matrices for each head, respectively. $\alpha^{\left(\mathrm{head}\right)}$ is the weight coefficient that governs the selective fusion of feature representations from different heads.

In traditional 3D medical image segmentation tasks, such as UNETR, the input resolution is typically downsampled from D×H×W to $\frac{D}{16}\times \frac{H}{16}\times \frac{W}{16}$ to reduce sequence length and enhance computational efficiency. However, this direct downsampling approach presents several limitations. It often results in a significant loss of detail in the input features, particularly restricting the expression of multi-scale features, which are crucial for accuracy in segmentation tasks. Capturing structural information at varying scales is essential for precise segmentation. Furthermore, the representation of global features may be insufficient, adversely affecting the model’s segmentation performance.

**Figure 5:**
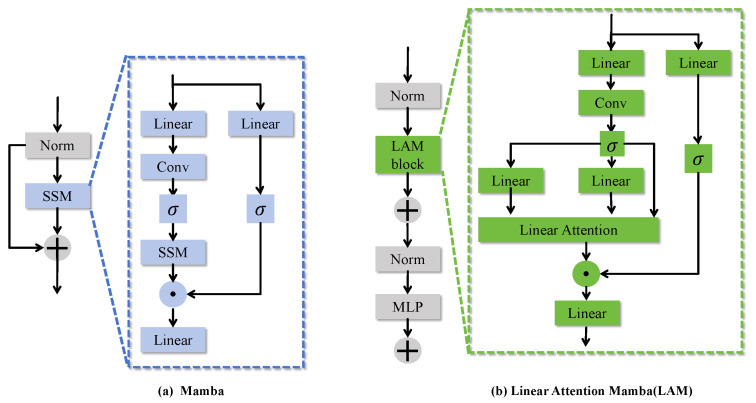
LAM structure diagram.

The linear attention mechanism(LAM) module builds upon the traditional Mamba framework by incorporating a linear attention mechanism to replace the conventional dense attention computation. This innovation significantly improves the computational efficiency of multi-scale feature modeling. As shown in Figure 5, LAM effectively captures the global information of multimodal features while maintaining the robust learning capacity of the traditional Mamba framework in handling complex multi-modal data. Consequently, it enhances the network’s adaptability and efficiency in processing large-scale datasets.

The core formula of the LAM is expressed as follows:

(10) $$Y^{\prime }=\mathrm{MLP}\left(X+\mathrm{Linear}\left(\mathrm{Attention}\left(Q,K,V\right)\right)\right)$$

By introducing a linear attention mechanism, LAM enhances feature representation while reducing computational complexity and memory requirements. Compared to traditional architectures, LAM effectively captures long-range dependencies, improving the model’s ability to handle large-scale data. By incorporating residual connections and a multilayer perceptron (MLP), LAM significantly enriches feature representation and improves the model’s generalization capabilities, all while maintaining computational efficiency.

### 3.3. RA Module

One effective approach for extracting boundary details is the incorporation of attention mechanisms. However, conventional methods often overlook the importance of adaptively leveraging multi-scale features to capture boundary details. To address this, we propose a hybrid domain attention mechanism designed to capture discriminative features with varying feature correlations, enabling more flexible feature learning and expression across different layers and dimensions.

**Figure 6:**
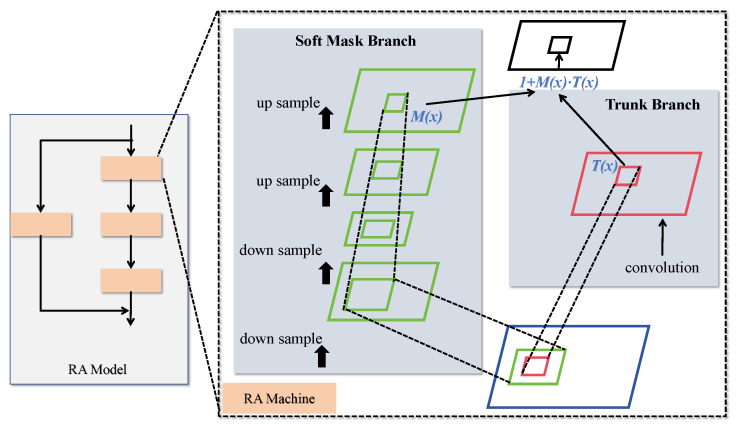
Structural diagram of RA.

The proposed hybrid attention mechanism, referred to as the residual attention (RA) structure, is illustrated in Figure 6. The RA module primarily consists of multiple attention units stacked through residual connections. Each attention module captures different types of information, allowing the model to extensively harness diverse forms of attention. This results in features with greater discriminative power. The RA mechanism effectively mimics a bottom-up feedforward process combined with top-down attention feedback within a single feedforward pass.

The hybrid attention module on the left in Figure 6 comprises a residual attention network built by stacking multiple attention modules. Each attention module consists of two branches: the trunk branch and the mask branch, as shown on the right side of Figure 3. The trunk branch performs feature processing, while the mask branch utilizes bottom-up and top-down structures to learn a mask of the same size. Given an input (*x*), the trunk branch produces an output (T(x)), and the mask branch outputs M(x), which serves as a soft weight for T(x). The mask controls the gating unit of the trunk branch, with the output of a typical attention mechanism module represented by the following equation:

(11) $$H_{i,c}\left(x\right)=M_{i,c}\left(x\right)*T_{i,c}\left(x\right)$$

Here, *i* denotes the spatial position, and *c* represents the channel. However, simply stacking attention modules can lead to performance degradation. Inspired by the ResNet architecture, where the soft mask is constructed as an identity mapping, the performance of the attention mechanism improves. Therefore, the output of the proposed enhanced hybrid attention mechanism is expressed as follows:

(12) $${\tilde{H}}_{i,c}\left(\begin{array}{c} x \end{array}\right)=\left(\begin{array}{c} 1+M_{i,c}\left(\begin{array}{c} x \end{array}\right) \end{array}\right)*T_{i,c}\left(\begin{array}{c} x \end{array}\right)$$

In this formulation, M(x) is constrained within the range of (0, 1], and F(x) represents the features generated by the network. When M(x) approaches 0, H(x) approximates F(x). The stacked attention module benefits from its incremental nature, enabling residual attention learning. By stacking multiple hybrid domain attention modules, the network’s representational capacity is progressively enhanced, allowing it to capture abstract features and relationships within the data more effectively.

The progressive improvement in the handling of multi-scale information is crucial for tasks such as building boundary extraction, as structures vary in size and complexity. The hybrid attention mechanism improves the model’s ability to capture fine boundary details, enhancing performance in multi-scale structural analysis.

### 3.4. Loss Function

In brain tumor image segmentation tasks, class imbalance is a common issue. Typically, the number of voxels representing normal tissues far exceeds that of tumor voxels. For instance, voxels for necrotic and enhancing tumor regions may constitute only a small fraction of the dataset, while the majority belong to normal tissue or non-enhancing tumor tissue. This imbalance poses specific challenges for the development of models aimed at automatic tumor segmentation. To mitigate the predictive bias caused by class imbalance, we designed a hybrid loss function combining Cross-Entropy Loss (CEL) and Generalized Dice Loss (GDL) to train the brain tumor segmentation model. The total loss function is formulated as follows:

(13) $$L=\left(1-\lambda \right)L_{g}+\lambda L_{c}$$

where λ is a weight coefficient balancing the two loss functions. Cross-entropy loss is effective for pixel-level classification tasks, emphasizing the importance of accurately classifying each pixel. The generalized Dice loss extends the traditional Dice loss by considering the size differences among classes, ensuring fairness across all categories. The cross-entropy loss is computed as follows:

(14) $$L_{c}=-\frac{1}{N}\sum_{i=1}^{N}\sum_{c=1}^{c}g_{ci}\log\left(p_{ci}\right)$$

The generalized Dice loss is defined as follows:

(15) $$L_{g}=1-\frac{2\sum_{c=1}^{C}\omega_{c}\sum_{i=1}^{N}p_{ci}g_{ci}}{\sum_{c=1}^{C}\omega_{c}\sum_{i=1}^{N}\left(p_{ci}+g_{ci}\right)}$$

Here, *C* represents the total number of classes, *N* is the total number of pixels, $p_{ci}$ is the predicted probability of the *i*-th pixel belonging to the *c*-th class, and $g_{ci}$ is the ground-truth label for the *i*-th pixel in the *c*-th class. The weight ($\omega_{c}$) for the *c*-th class is typically set as the inverse frequency of the class to address the class imbalance issue effectively.

## 4. Experimental Results and Analysis

### 4.1. Dataset

The Brain Tumor Segmentation Challenge (BraTS Challenge) is one of the most prestigious and long-standing competitions organized by the Medical Image Computing and Computer-Assisted Intervention Society (MICCAI). Having been held annually for over a decade, it remains a cornerstone in the field of medical image processing. Each BraTS case includes four modalities of magnetic resonance imaging (MRI), with each modality having dimensions of 240 × 240 × 155 (L × W × H).

The four MRI modalities are described as follows:

T1: This modality is primarily used to observe anatomical structures, although lesions are not displayed as clearly.

T1ce: After injecting a contrast agent into the bloodstream, this modality highlights regions with active blood flow, serving as a critical indicator for enhancing tumors.

T2: This modality provides clearer visualization of lesions, aiding in the overall assessment of the tumor.

FLAIR: This modality highlights regions with high water content, which is particularly useful for identifying peritumoral edema.

For this study, we selected the BraTS 2020 [26] and BraTS 2023 [27] datasets.

### 4.2. Experimental Setup

#### 4.2.1. Hardware Configuration

The entire experimental process was conducted on a system running Ubuntu 20.04. The hardware specifications include the following: an Intel i7-9700 processor, an NVIDIA RTX A5000 GPU with 24 GB of VRAM, and 32 GB of RAM.

#### 4.2.2. Software Configuration

The experiments were implemented using the Python-3.9 programming language, with Anaconda employed for environment management. Visual Studio Code (VSCode-1.5) was chosen as the Integrated Development Environment (IDE). The key third-party libraries utilized in this study include the following: PyTorch1.18.0, a deep learning framework used for model training; SimpleITK-2.5.0, an open-source, cross-platform library for image processing, particularly for the reading of MRI image data; and NumPy1.22.4, which was used for numerical computations.

#### 4.2.3. Experimental Settings

The Adam optimizer was used for training, with the batch size set to 8. The initial learning rate was configured at 0.001 and gradually decreased as the number of training epochs increased. The loss function combined Dice loss and cross-entropy loss to balance segmentation performance.

### 4.3. Evaluation Metrics

Evaluation metrics provide a quantitative method to assess and measure the performance of the segmentation approach. In this study, the segmentation effectiveness is evaluated both qualitatively and quantitatively.

Qualitative Evaluation: Qualitative evaluation involves visualizing the segmented brain tumor images to observe the segmentation quality. This assessment is primarily based on human visual perception, allowing for a subjective understanding of the segmentation performance.

Quantitative Evaluation: For quantitative evaluation, two specific metrics are employed: the Dice similarity coefficient (Dice) and the Hausdorff distance (HD).

The Dice coefficient measures the overlap between the predicted segmentation and the ground truth at the pixel or voxel level, reflecting the model’s accuracy in classifying pixels or voxels correctly. It is commonly used in evaluating medical image segmentation. The Dice coefficient ranges from 0 to 1, where a value closer to 1 indicates a higher accuracy and a value of 1 signifies a perfect match between the segmentation and the ground truth. The formula for calculating the Dice coefficient is expressed as follows:

(16) $$Dice=\frac{2*|A\cap B|}{\left(|A|+|B|\right)}$$

Here, *A* represents the predicted segmentation; *B* represents the actual segmentation; |A∩B| denotes the area of overlap between *A* and *B*; and |A| and |B| denote the areas of *A* and *A*, respectively.

The Hausdorff distance quantifies the maximum discrepancy between two sets of points and is widely used in medical image segmentation to measure the worst-case boundary deviation between predicted and ground-truth segmentations. For two point sets (*A* and *B*), the Hausdorff distance (HD) is defined as follows:

(17) $$HD\left(A,B\right)=max\left\{h(A,B),h(B,A)\right\}$$

Here, h(A,B) denotes the directed Hausdorff distance from set *A* to set *B*, defined as follows:

(18) $$h\left(A,B\right)=\max_{a\in A}\min_{b\in B}\left|a-b\right|$$

This formulation captures the greatest distance from a point in set *A* to the closest point in set *B* and vice-versa for h(A,B). Thus, the overall HD reflects the largest boundary discrepancy between the two segmentations.

### 4.4. Ablation Study

In this section, detailed ablation experiments are conducted to verify the effectiveness of each module in the proposed method. The results of the ablation study are shown in Table 1. We progressively removed the multimodal data processing module, the Mamba-like module, and the attention-like module, and evaluated the model’s performance using the Dice coefficient [28,29] and Hausdorff distance (HD).

Method 1: Without any multimodal data processing or attention mechanisms, the baseline model exhibited average performance, with a mean Dice of 77.98% a mean HD of 25.99%. This result indicates that a simple convolutional network cannot fully utilize multimodal information.

Method 2: After adding the TB-TMA module, the model’s performance improved, with the mean Dice increasing to 79.34% and the mean HD decreasing to 23.6%. The TB-TMA module effectively enhanced the extraction and fusion of multimodal features.

Method 3: Introducing the SSM module alone resulted in a mean Dice of 78.83% and mean HD of 25.15%. While the SSM module improved feature extraction to some extent, its effectiveness was slightly lower compared to the TB-TMA module.

Method 4: When both the TB-TMA and SSM modules were introduced simultaneously, the model’s mean Dice increased to 81.45%, and the mean HD decreased to 22.47%. This indicates that the synergistic effect of both modules significantly enhanced the model’s performance.

Method 5: Replacing the SSM module with the SegMamba module led to a further increase in the mean Dice to 82.65% and a decrease in the mean HD to 21.69%. The SegMamba module demonstrated stronger capabilities in multimodal feature modeling.

Method 6: Introducing the LAM module on top of the TB-TMA module, the mean Dice reached 83.67%, and the mean HD decreased to 20.81%. The LAM module significantly improved computational efficiency and feature modeling through linear attention mechanisms.

Method 7: Adding cross-attention on top of the LAM module further increased the mean Dice to 84.89% and reduced the mean HD to 19.79%. Cross-attention effectively enhanced the interaction between different modality features.

Method 8: Incorporating the TransBTS module resulted in a substantial improvement, with the mean Dice rising to 86.47% and the mean HD dramatically decreasing to 9.11%. The TransBTS module significantly enhanced segmentation performance through deeper feature extraction.

Method 9: Finally, employing the residual attention (RA) module yielded the highest mean Dice of 87.66% and the lowest mean HD of 8.92%. The RA module significantly improved the capture of boundary details through hybrid domain attention mechanisms.

The experimental results on the BraT2023 dataset were consistent with those on the BraT2020 dataset, as shown in Table 2. Although the specific values varied, the performance improvement trend for each module remained similar, further validating the robustness and effectiveness of the proposed method across different datasets.

**Figure 7:**
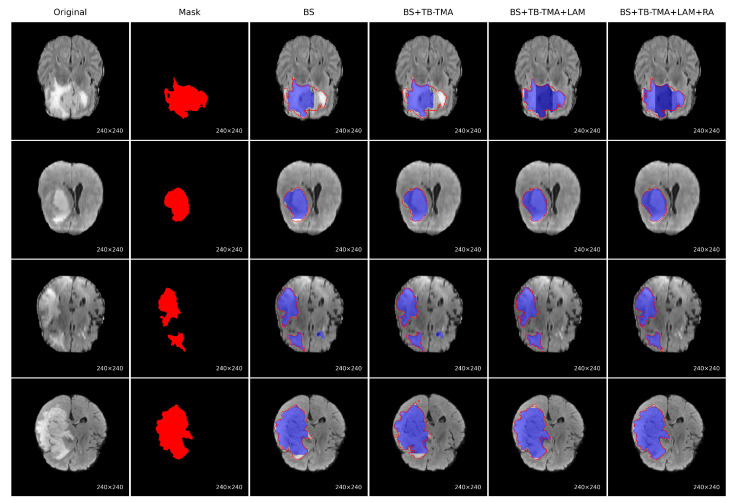
Visual comparison of ablation results on the BraTS brain tumor segmentation task. Each row represents a sample, showing the following (from **left** to **right**): the original image; ground-truth mask; baseline (BS) output; BS with TB-TMA module; BS with TB-TMA and LAM modules; and the final model with TB-TMA, LAM, and RA modules. The progressive improvements demonstrate the effectiveness of each proposed component in enhancing segmentation completeness and boundary accuracy. In the Mask column, the red regions represent the true tumor segmentation. In all predicted results, the blue areas indicate the predicted segmentation, while the red contour lines overlay the ground-truth boundaries for comparison.

To evaluate the performance improvement of each module, we conducted a series of ablation experiments on the BraTS brain tumor segmentation task, with visual results presented for each approach, as shown in Figure 7. The baseline model (BS) showed significant issues, such as missing tumor regions and inaccurate boundaries. After incorporating the TB-TMA module, the model effectively fused unimodal and cross-modal features through a dual-branch structure and cross-attention mechanism, enhancing the model’s cross-modal representation ability and resulting in more complete tumor segmentation. Further integration of the LAM module improved the model’s multi-scale feature modeling capability, leading to better identification of large tumor areas. Finally, the RA module, with its residual structure and attention stacking, refined boundary segmentation and significantly improved the precision of edge region localization. The combined results demonstrate that the synergistic effect of these modules significantly enhances both accuracy in segmenting tumor regions and boundary detail restoration.

### 4.5. Comparative Experiments

In this section, we present a detailed comparison of the proposed method with several mainstream brain tumor segmentation models. The experimental results are shown in Table 3. To provide a comprehensive analysis of the superiority of our approach, we compare its performance with that of other methods on the BraTS2020 dataset using two key metrics: the Dice coefficient and the Hausdorff distance (HD), covering three regions: whole tumor (WT), tumor core (TC), and enhancing tumor (ET). First, from a quantitative perspective, our method demonstrates significant performance improvements in terms of both the Dice coefficient and the Hausdorff distance (HD) metrics.

Specifically, for the whole tumor (WT) region, the Dice coefficient of our method reaches 92.47%, which represents an improvement of approximately 8.36% over 3D U-Net and surpassing V-Net and SegTransVAE by notable margins. This improvement highlights the enhanced precision of our method in capturing the entire tumor region. In the tumor core (TC) region, our method achieves a Dice coefficient of 87.19%, which marks a 15.8% improvement over V-Net and a 14.3% improvement over SegTransVAE. Particularly in the enhancing tumor (ET) region, our method performs exceptionally well, achieving a Dice coefficient of 81.32%, which is an increase of approximately 12.56% compared to 3D U-Net and around 31.7% higher than V-Net. This result demonstrates the significant advantage of our method in segmenting more challenging regions, especially the enhancing tumor area.

In addition to improvements in the Dice coefficient, the Hausdorff distance (HD) is another critical evaluation metric. Our method achieves a Hausdorff distance of 4.01 mm in the WT region, which is approximately 70% lower than 3D U-Net and 80% lower than V-Net, clearly showcasing the superior boundary precision of our approach. Similarly, in the TC region, our method’s HD is 5.18 mm, which represents a reduction of about 62% compared to 3D U-Net and 57% compared to V-Net. In the ET region, our method achieves a Hausdorff distance of 15.55 mm, which is about 69% lower than 3D U-Net and 67% lower than V-Net. These results indicate that our method significantly outperforms others in accurately capturing tumor boundaries, especially when handling complex boundaries.

Overall, the proposed method outperforms existing mainstream approaches in terms of both the Dice coefficient and the Hausdorff distance across multiple tumor regions, with particularly notable performance improvements in the enhancing tumor region. Compared to traditional models like 3D U-Net and V-Net, our method achieves substantial advancements in segmentation accuracy and boundary precision, which are crucial for tumor localization and treatment in clinical applications. These results fully demonstrate the potential and advantages of our approach in brain tumor segmentation tasks.

Table 4 presents a comparison of different brain tumor segmentation methods on the BraTS2023 dataset, evaluating performance using the Dice coefficient and Hausdorff distance (HD) for the whole tumor (WT), tumor core (TC), and enhancing tumor (ET) regions. The proposed method outperforms other approaches, achieving a Dice coefficient of 88.07%, which is notably higher than that of TransBTSv2. Specifically, it excels in the TC (88.5%) and ET (82.5%) regions. Our method achieves an HD of 3.8 mm for WT and 13.8mm for ET, both lower than TransBTSv2’s 4 mm and 15 mm, respectively, indicating superior boundary precision. Overall, our method demonstrates significant improvements in segmentation accuracy and boundary fitting, particularly in the ET region, outperforming current state-of-the-art methods and showcasing its potential in brain tumor segmentation.

Table 5 presents a comprehensive comparison of various brain tumor segmentation methods on the BraTS2020 dataset, focusing on both segmentation performance and model complexity. Specifically, it includes two primary performance metrics: the Dice coefficient and Hausdorff Distance (HD), which assess segmentation accuracy and boundary precision, respectively. In addition, two complexity metrics, namely floating-point operations (FLOPs, in GigaFLOPs) and the number of parameters (in millions),are reported to evaluate the computational burden and model size, respectively. This enables a multi-dimensional analysis of each method’s trade-off between accuracy and efficiency.

In terms of segmentation performance, the three variants of our proposed method—Ours (TB-TMA), Ours (TB-TMA + LAM), and Ours (TB-TMA + LAM + RA)—consistently achieve competitive accuracy while maintaining low computational complexity. The full model, Ours (TB-TMA + LAM + RA), attains a Dice score of 87.66% and an HD of 8.92 mm, ranking highest among all compared methods. Compared with the advanced lightweight model, TransBTSv2, our method improves segmentation accuracy by 2.76% and boundary accuracy by 10.17% while simultaneously reducing FLOPs by 24.5% and the parameter count by 29.3%, highlighting the significant advantage in terms of model compactness.

Furthermore, the progressive enhancement from the baseline model (TB-TMA) to the full version through the integration of the LAM and RA modules leads to consistent performance gains. Specifically, the Dice score improves from 79.34% to 83.67% and, finally, 87.66%, while the HD decreases from 19.37 mm to 13.28 mm, then to 8.92 mm. These improvements validate the effectiveness of the proposed modules, demonstrating their critical roles in enhancing both segmentation accuracy and boundary delineation.

From the perspective of model complexity, although several Transformer-based architectures (e.g., UNETR and 3DUXNET) achieve good segmentation performance, they suffer from substantially higher computational costs. For instance, UNETR requires 58.7 GFLOPs and over 100 million parameters, which poses significant challenges for deployment in real-world clinical environments. In contrast, our full model requires only 1.42 GFLOPs and 10.83 million parameters, greatly reducing the dependency on computational resources and showcasing strong practicality and deployability.

In summary, the proposed method demonstrates superior performance across multiple dimensions. It achieves high segmentation accuracy with significantly reduced model complexity. The validated effectiveness of the proposed modules, coupled with the lightweight nature of the overall architecture, makes our approach highly promising for real-world clinical applications.

### 4.6. Algorithm Visualization

**Figure 8:**
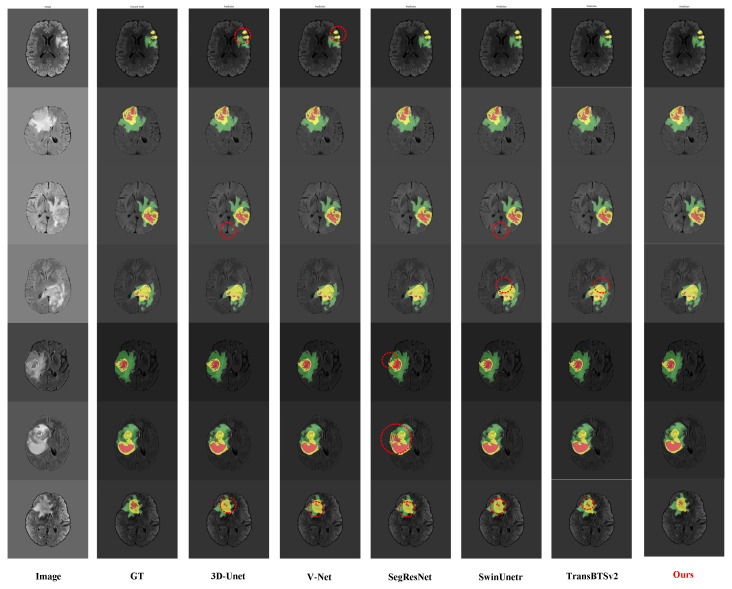
Performance comparison of different methods on the BraTS2020 dataset. Red dashed circles indicate regions with significant segmentation differences among methods, highlighting the effectiveness of the proposed approach in challenging areas. The segmentation results use different colors to represent tumor regions: green for the whole tumor (WT), yellow for the tumor core (TC), and red for the enhancing tumor (ET).

Figure 8 demonstrates the segmentation performance of our proposed algorithm on the BraTS2020 dataset for medical tumor images. Each set of images, from left to right, includes the original image, ground-truth annotations, and the model’s predicted results. To visually distinguish the segmentation outcomes, we used different colors to label various tumor regions: green for the whole tumor (WT), yellow for the tumor core (TC), and red for the enhancing tumor (ET). Furthermore, red dashed circles are used to highlight regions where the segmentation results between different methods exhibit noticeable differences. These marked areas help emphasize the effectiveness of our method in handling challenging and irregular tumor shapes. The visualizations clearly show that our method can accurately identify and segment different types of tumor regions, closely matching the ground-truth annotations, which highlights its superior segmentation performance. Such detailed segmentation is crucial for clinical diagnosis and treatment planning.

The comparative visualization below illustrates the segmentation results of our proposed algorithm alongside several mainstream methods on the BraTS2020 dataset. Specifically, from left to right, the images show the original image, ground truth, results from 3D U-Net, V-Net, SegResNet, SwinUNETR, TransBTSv2, and our proposed method. Our algorithm excels in capturing tumor boundary details, especially in complex regions with irregular shapes or fuzzy boundaries. In contrast, other methods may exhibit mis-segmentations or blurred boundaries in some challenging areas.

For the whole tumor (WT), tumor core (TC), and enhancing tumor (ET) regions, our algorithm consistently delivers high segmentation accuracy across different images. Notably, in the segmentation of the enhancing tumor region (red), our method shows exceptional performance in capturing internal lesions, which is critical for clinical diagnosis. Other methods occasionally struggle with under-segmentation or over-segmentation in this region. The comparative results across different image samples demonstrate that our method maintains effective and stable segmentation across various tumor morphologies. This indicates strong generalization capability, making our algorithm well-suited for the handling of diverse and complex scenarios in real-world applications.

## 5. Conclusions

This paper introduces an advanced multimodal deep learning framework for brain tumor segmentation, integrating the two-branch two-model attention (TB-TMA); linear attention Mamba (LAM); and residual attention (RA) modules to enhance multimodal feature extraction, attention modeling, and boundary refinement. Experimental results on the BraTS2020 and BraTS2023 datasets show that our method achieves Dice scores of 87.66% and 88.07%, respectively, and significantly reduces the Hausdorff distance to as low as 7.4mm, on average, outperforming methods such as TransBTSv2. Although the hybrid network proposed in this paper has achieved remarkable results in multimodal brain tumor segmentation, it still has some shortcomings. For some complex or irregularly shaped tumors, there is still room for further improvement in segmentation accuracy. In addition, existing models still face challenges in terms of computational efficiency and reasoning speed when processing large-scale medical images. In future work, we plan to further optimize the existing methods and improve the expression and learning efficiency of the network by introducing more advanced network architectures. We will also explore adaptive multi-scale feature modeling methods to better address the diversity of tumor morphology. In addition, we hope to be able to extend the model to more image modes, such as PET and CT scans, to improve the applicability of the model in different clinical settings.

## Figures and Tables

**Table 1:** Performance comparison of different methods (BraT2020 dataset). Dice coefficient indicates segmentation accuracy (higher is better), and HD (Hausdorff distance) indicates boundary alignment (lower is better).

Method	Multimodal Data Processing	Mamba-Like Module	Attention-Like Module	Dice (%)				HD (%)			
				WT	TC	ET	Mean	WT	TC	ET	Mean
1	×	×	×	84.11	79.06	68.76	77.98	13.366	13.607	50.983	25.99
2	TB-TMA	×	×	86.45	81.23	70.34	79.34	12.478	12.103	46.213	23.6
3	×	SSM	×	85.22	80.17	69.11	78.83	13.132	12.984	49.345	25.15
4	TB-TMA	SSM	×	88.37	83.29	72.68	81.45	11.204	11.456	44.762	22.47
5	TB-TMA	SegMamba	×	89.56	84.51	73.89	82.65	10.739	10.803	43.526	21.69
6	TB-TMA	LAM	×	90.23	85.67	75.12	83.67	9.786	10.294	42.351	20.81
7	TB-TMA	LAM	Cross-Attention	91.15	86.49	77.04	84.89	8.765	9.832	40.765	19.79
8	TB-TMA	LAM	TransBTS	92.01	87.05	80.34	86.47	4.685	5.658	16.975	9.11
9	TB-TMA	LAM	RA	92.47	87.19	81.32	87.66	4.013	5.184	15.55	8.92

Note: “×” denotes that the corresponding module is not used in this configuration.

**Table 2:** Ablation experiment (BraT2023 dataset).

Method	Multimodal Data Processing	Mamba-Like Module	Attention-Like Module	Dice(%)				HD(%)			
				WT	TC	ET	Mean	WT	TC	ET	Mean
1	×	×	×	85.23	80.34	69.82	78.46	12.654	12.905	48.769	24.77
2	TB-TMA	×	×	87.58	82.41	71.45	80.48	11.932	11.673	45.127	22.91
3	×	SSM	×	86.47	81.29	70.23	79.33	12.635	12.493	47.458	24.2
4	TB-TMA	SSM	×	89.72	84.76	73.52	82.67	10.679	10.922	43.245	21.62
5	TB-TMA	SegMamba	×	90.89	85.89	74.75	83.84	10.194	10.367	42.081	20.88
6	TB-TMA	LAM	×	91.57	86.94	76.02	84.84	9.279	9.849	40.927	20.02
7	TB-TMA	LAM	Cross-Attention	92.43	87.72	78.24	86.13	8.305	9.351	39.427	19.03
8	TB-TMA	LAM	TransBTS	93.31	88.28	81.15	87.58	4.295	5.138	15.841	8.43
9	TB-TMA	LAM	RA	93.78	88.43	82.12	88.11	3.679	4.679	14.721	7.69

Note: “×” denotes that the corresponding module is not used in this configuration.

**Table 3:** Performance comparison of different methods (BraT2020 dataset).

Method	Dice (%)				HD (mm)			
	WT	TC	ET	Mean	WT	TC	ET	Mean
3D Unet [9]	84.11	79.06	68.76	77.31	13.366	13.607	50.983	25.99
V-Net [10]	84.63	75.26	61.79	73.89	20.407	12.175	47.702	26.76
SegTransVAE [30]	85.5	76.3	64	75.27	19.5	12	45.5	25.67
SegResNet [31]	86.1	78.5	68.2	77.6	12.3	10.8	40.5	21.2
Attention Unet [32]	87.5	80.6	70.9	79.67	10.2	9.6	35.4	18.4
UNETR [14]	88	81	72.5	80.5	9.5	8.9	33.8	17.4
SwinUNETR [15]	89	82.5	75	82.17	7.8	7.5	30.6	15.3
3DUXNET [33]	89.5	83.2	76.8	83.17	6.5	6.8	25.7	12.33
TransBTS [34]	90.09	81.73	78.73	83.52	4.964	9.769	17.947	10.89
TransBTSv2 [17]	90.56	84.5	79.63	84.9	4.272	5.56	17.947	9.93
Our method	92.47	87.19	81.32	87.66	4.013	5.184	15.55	8.92

**Table 4:** Performance comparison of different methods (BraT2023 dataset).

Method	Dice (%)				HD (mm)			
	WT	TC	ET	Mean	WT	TC	ET	Mean
3D Unet	85.5	80.5	70.2	78.73	12.5	12.9	48.5	24.63
V-Net	85.9	77.8	64.5	76.73	19	11.5	45.8	25.43
SegTransVAE	86.8	79.2	67.8	77.93	18.2	11.2	43	24.13
SegResNet	87.5	80.9	70	79.47	11.8	10	38.2	20.67
Attention Unet	88.9	82.3	72.5	81.23	9.8	8.7	32.7	17.73
UNETR	89.5	83	74.3	82.93	8.9	8.2	30.4	15.83
SwinUNETR	90.5	84	76.5	83.67	7.2	6.8	27.2	13.73
3DUXNET	91	85	78.3	84.77	5.8	5.6	23.5	11.63
TransBTS	91.3	83.5	79.5	84.77	4.8	9	15.9	9.9
TransBTSv2	91.8	86	80	85.93	4	5	15	8
Our method	93.2	88.5	82.5	88.07	3.8	4.6	13.8	7.4

**Table 5:** Model complexity comparison on the BraT2020 dataset.

Method	Dice	HD	FLOPs (G)	Params (M)
3D Unet	77.31	25.99	13.04	16.21
V-Net	73.89	26.76	5.85	45.61
SegTransVAE	75.27	25.67	7.41	44.7
SegResNet	77.6	21.2	3.18	13.5
Attention Unet	79.67	18.4	18.3	23.8
UNETR	80.5	17.4	58.7	101
SwinUNETR	82.17	15.3	1.96	27.15
3DUXNET	83.17	12.33	22.1	31.5
TransBTS	83.52	10.89	2.6	32.99
TransBTSv2	84.9	9.93	1.88	15.3
Ours (TB-TMA)	79.34	19.37	1.08	8.91
Ours (TB-TMA + LAM)	83.67	13.28	1.22	9.38
Ours (TB-TMA + LAM + RA)	87.66	8.92	1.42	10.83

## Data Availability

The raw data supporting the conclusions of this article will be made available by the authors upon request.
